# Supplementary material for: Prevalence and associated factors of anemia during pregnancy at a tertiary hospital in Zambia
Source: PLoS One. 2025 Jul 17;20(7):e0327801. doi: 10.1371/journal.pone.0327801 (PMC12270163; doi:10.1371/journal.pone.0327801)
Supplement: Attachment — Submitted filename: responses to the reviewers.docx [file pone.0327801.s004.docx]

24^th^ December, 2024

To the reviewers and Editor,

**Ref: RESPONSES TO REVIEWER COMMENTS**

We would like to thank the reviewers for taking the time to make suggestions that have improved our manuscript. We have now made revisions to the comments in the manuscript and incorporated all suggestions. We now hope the current manuscript is acceptable for publication. Below are the point-by-point responses to all comments and suggestions

**Editorial comments**

1. Please ensure that your manuscript meets PLOS ONE's style requirements, including those for file naming. The PLOS ONE style templates can be found at

<https://journals.plos.org/plosone/s/file?id=wjVg/PLOSOne_formatting_sample_main_body.pdf> and 
<https://journals.plos.org/plosone/s/file?id=ba62/PLOSOne_formatting_sample_title_authors_affiliations.pdf>

**Response: thank you. Files named according**

2. Please amend your list of authors on the manuscript to ensure that each author is linked to an affiliation. Authors’ affiliations should reflect the institution where the work was done (if authors moved subsequently, you can also list the new affiliation stating “current affiliation:….” as necessary).

**Response: thank you. all authors have been given an affiliations**

3. Please ensure that you refer to Figure 1 in your text as, if accepted, production will need this reference to link the reader to the figure.

**Response: thank you. Figure have been removed and uploaded as separate figures acceptable to the Journal requirements**

4. Please include captions for your Supporting Information files at the end of your manuscript, and update any in-text citations to match accordingly. Please see our Supporting Information guidelines for more information: <http://journals.plos.org/plosone/s/supporting-information>.

**Response: thank you.** Supporting document caption has been updated at the end of the manuscript as per journal guidelines

5. We note that there is identifying data in the Supporting Information file <S2_minimal data set.xlsx>. Due to the inclusion of these potentially identifying data, we have removed this file from your file inventory. Prior to sharing human research participant data, authors should consult with an ethics committee to ensure data are shared in accordance with participant consent and all applicable local laws.

Data sharing should never compromise participant privacy. It is therefore not appropriate to publicly share personally identifiable data on human research participants. The following are examples of data that should not be shared:

-Name, initials, physical address
-Ages more specific than whole numbers
-Internet protocol (IP) address
-Specific dates (birth dates, death dates, examination dates, etc.)
-Contact information such as phone number or email address
-Location data
-ID numbers that seem specific (long numbers, include initials, titled “Hospital ID”) rather than random (small numbers in numerical order)

Data that are not directly identifying may also be inappropriate to share, as in combination they can become identifying. For example, data collected from a small group of participants, vulnerable populations, or private groups should not be shared if they involve indirect identifiers (such as sex, ethnicity, location, etc.) that may risk the identification of study participants.

Additional guidance on preparing raw data for publication can be found in our Data Policy (<https://journals.plos.org/plosone/s/data-availability#loc-human-research-participant-data-and-other-sensitive-data>) and in the following article: <http://www.bmj.com/content/340/bmj.c181.long>.

Please remove or anonymize all personal information (Age in years) ensure that the data shared are in accordance with participant consent, and re-upload a fully anonymized data set. Please note that spreadsheet columns with personal information must be removed and not hidden as all hidden columns will appear in the published file.

**Response:** thank you. All identifying information have been removed from the data set

6. Please review your reference list to ensure that it is complete and correct. If you have cited papers that have been retracted, please include the rationale for doing so in the manuscript text, or remove these references and replace them with relevant current references. Any changes to the reference list should be mentioned in the rebuttal letter that accompanies your revised manuscript. If you need to cite a retracted article, indicate the article’s retracted status in the References list and also include a citation and full reference for the retraction notice.

**Response:** thank youall references have been checked and cited accordingly in the text.

**Reviewer Comments**

**Reviewer #1**: The manuscript addresses a very important and common problem. It is well written.

**Response:** thank you for reviewing of our article

My only comment is related to the demographic data. You included religion, does being protestant or catholic has different diet that could affect the prevalence of anaemia. If not please remove it. The same goes for the residence.

**Response:** Thank you very much. We included religion because, in our setting, certain Protestant religious groups (for example, Seventh-day Adventists, who make up 7.1%) advocate for vegetarianism. Regarding residence, we aimed to explore whether differences exist, as rural-based individuals tend to have a predominantly vegetable-based diet when compared to the urban based individuals.

Also please add the word complications to the (history of antepartum and history of postpartum)

**Response:** thank you for the suggestion. We have added the word complication

**Reviewer #2:**

There is some grammar mistakes that the authors must correct.

**Response:** thank you for the observation. We have made effort to revise grammar.

The mathematical equations of presented model were not provided.

**Response:** Thank you. To arrive at the final model, we used variables with p-values less than 0.20 and those supported by the literature. These were included in the multivariable analysis, as explained in the methods section.

A figure that depicted the anemia in terms of ages were not presented.

**Response:** Thank you. A figure depicting anemia in terms of age has now been presented as Figure 2.

We have revised the manuscript and addressed all concerns raised by the reviewers. We want to thank you all again for the tremendous work and time that you committed in editing our work. Our manuscript is much improved, and we are very grateful.

Yours sincerely,

Dr. Lukundo Siame, Bsc., MBcHB.

Junior Residence Medical officer Livingstone University Teaching Hospital, Zambia 0
