# Supplementary material for: Prevalence and associated factors of anemia during pregnancy at a tertiary hospital in Zambia
Source: PLoS One. 2025 Jul 17;20(7):e0327801. doi: 10.1371/journal.pone.0327801 (PMC12270163; doi:10.1371/journal.pone.0327801)
Supplement: Attachment — Submitted filename: Response to Reviewers.docx [file pone.0327801.s005.docx]

16/06/2025

PLOS ONE Journal

Dear Editor,

**Ref: Submission of a revised research article for peer review and publication consideration**

Reference to the above-mentioned subject. I am writing to submit a revised original research article titled "Prevalence and Associated Factors of Anemia During Pregnancy at Livingstone University Teaching Hospital in Zambia: A Retrospective Cross-Sectional Study,".

We would like to thank the reviewers for taking the time to make suggestions that have improved our manuscript. We have revised the manuscript and addressed all concerns and suggestions. We now hope the current manuscript is acceptable for publication. Below are the point-by-point responses to all comments and suggestions.

**Response to the reviewer:**

**Academic editor’s comment:**

Thank you for this revision. I am a new editor recently assigned to this manuscript. I have just one last editing note: In the Results section, please do not begin sentences with a number, e.g., 8.2% of women xxx etc. You can begin those sentences with the spelling of the number, e.g., Eight percent of women xxx etc. or reconstruct those sentences, e.g., Few women (8.2.%) were xxx etc.

**Response**: Thank you very much for the suggestion: we have revised the manuscript the manuscript as suggested.

**Reviewer comments:**

Reviewer #2: The answers to all comments were presented and prepared. There are no any reminded comments to be answered.

**Response**: thank you.

We have revised the manuscript and addressed all concerns raised. We want to thank you all again for the tremendous work and time that you committed to reviewing and correcting our work. Our manuscript is much improved, and we are very grateful.

Please address all correspondence to [lukundosiame23@gmail.com](mailto:lukundosiame23@gmail.com) . We look forward to hearing from you at your earliest convenience.

Please do not hesitate to contact me should you have further questions.

Yours sincerely,

Dr. Lukundo Siame, Bsc., MBcHB.

Junior Residence Medical Livingstone University Teaching Hospital
